# Influence of Renin-Angiotensin System Inhibitors on Postoperative Delirium in Patients With Pulmonary Arterial Hypertension: A Secondary Analysis of a Retrospective Cohort Study

**DOI:** 10.3389/fpsyt.2022.851104

**Published:** 2022-04-08

**Authors:** Gong Chen, Sai Zhou, Fang Deng

**Affiliations:** ^1^Department of Anesthesiology, The Third Xiangya Hospital of Central South University, Changsha, China; ^2^Academic Affairs Department, Changsha Medical University, Changsha, China; ^3^Department of Anesthesiology, Xiangya Changde Hospital, Changde, China

**Keywords:** postoperative delirium, pulmonary arterial hypertension, ACEIs, ARBs, renin-angiotensin system inhibitors

## Abstract

**Objective:**

To investigate the correlation between preoperative use of ACEIs/ARBs and postoperative delirium (POD) in surgical patients with pulmonary arterial hypertension (PAH).

**Methods:**

The present study is a secondary analysis of a retrospective cohort study conducted at the University of Washington Medical Center from April 2007 to September 2013. Patients with PAH who underwent non-cardiac, non-obstetric surgery were enrolled in the original research. We further excluded stroke, sepsis, and craniotomy patients from interfering with POD evaluation. The univariate regression analysis and multivariate-adjusted model were used to explore the influence of preoperative ACEIs/ARBs use on the occurrence of POD.

**Results:**

A total of 539 patients were included in this study. The incidence of POD in these patients was 3.0%. Following the adjustment of potential confounders (age, BMI, smoking status, pulmonary arterial systolic pressure, length of surgery, vascular surgery, asthma, obstructive sleep apnea, renal failure, atrial fibrillation, coronary artery disease, hydrochlorothiazide, alpha-blocker, calcium channel blocker, antiplatelet, steroids, statin, isoflurane), a negative relationship was found between preoperative use of ACEIs/ARBs and occurrence of POD (OR = 0.15, 95%CI: 0.03 to 0.80, *P* = 0.0266).

**Conclusion:**

Preoperative use of ACEIs/ARBs in patients with PAH reduces the risk of POD. ACEIs/ARBs may be more recommended for patients with PAH in the future.

## Introduction

Postoperative delirium (POD) is a clinical syndrome characterized by acute and fluctuating consciousness, attention, and cognition alterations after surgery (1). It is the most common neurological complication after surgery, with incidences varying between 10 and 45% (1, 2). In addition, it is associated with increased morbidity and mortality (3). Previous studies reported that patients with cognitive impairment before surgery were at higher risk of POD (4). At the same time, some drugs that can delay cognitive decline also have POD-preventing properties, such as non-steroidal anti-inflammatory drugs (5, 6).

Renin-angiotensin system (RAS) inhibitors are widely used for the management of hypertension, which includes angiotensin-converting enzyme inhibitors (ACEIs) and angiotensin receptor blockers (ARBs). Except for anti-hypertension, ACEIs and ARBs show a beneficial effect in slowing the cognitive impairment associated with Alzheimer's disease (7). In addition, it was reported that the patients who received ACEIs/ARBs during the postoperative intensive care unit had a lower risk of POD (8). However, we do not know whether preoperative use of ACEIs/ARBs benefits POD prevention.

The current study performed a secondary analysis of published data. In the original article, Shah et al. studied postoperative outcomes in patients with pulmonary arterial hypertension (PAH) (9). The population with PAH may be particularly at risk to POD since many factors that lead to PAH are risk factors for POD, such as smoking, COPD, valvular disease, and pulmonary circulation disorders (10–12). Interestingly, the original data contains valuable information about ACEIs/ARBs medication and POD. Using the original data, we aim to explore the correlation between preoperative use of ACEIs/ARBs and POD in surgical patients with PAH. We hypothesize that PAH patients who take ACEIs/ARBs preoperatively have a lower risk of POD.

## Methods

### Study Design

The original research was a retrospective cohort study approved by the Institutional Review Board of the University of Washington after waiving the requirement for informed consent (9). The current work is a secondary analysis of the original data. All the studies described in the paper were in line with the Declaration of Helsinki promulgated by the National Institute of Health.

### Inclusion and Exclusion Criteria

Shah et al. performed the original study. Briefly, they initially reviewed 1,922 adult patients with PAH (diagnosed from previous cardiac catheterization or echocardiography) who received general anesthesia for elective surgery from April 2007 to September 2013. The inclusion criteria must also meet: (1) The cardiac catheterization or echocardiography examination data must be obtained within 1 year before the operation; (2) The elective surgery must be non-cardiac surgery and non-obstetric surgery (9). On this basis, the present study further excluded stroke, sepsis, and craniotomy patients to not interfere with POD evaluation. Finally, 539 patients were included in this study.

### Measurements of Covariates and Outcome

The measurements of the covariates and outcomes were described in detail in the original. Briefly, preoperative covariates included baseline demographics (age, sex, body mass index (BMI), ASA classification, functional status, smoking status), comorbid illnesses (angina, renal failure, diabetes, obstructive sleep apnea, asthma, coronary artery disease, arrhythmia, PAH), preoperative medication (statin, steroids, antiplatelet, anticoagulant, anti-hypertension drugs). This preoperative information was acquired from the record of the pre-anesthesia clinic. Since the study population was the patients with PAH, we obtained some indicators related to PAH in cardiac catheterization or echocardiogram examinations from the database. The intraoperative factors included surgery-related factors (length of surgery, open surgical approach, intraabdominal surgery, intrathoracic surgery, vascular surgery), and intraoperative medication (inhalational anesthetic agents, atropine). The intraoperative factors were acquired from the intraoperative anesthesia record.

The primary outcome is the occurrence of POD. The gold standard for diagnosing POD is the Diagnostic and Statistical Manual of Mental Disorders, 5th edition (DSM5) (13). However, no information was available in the original database on which tools were used to assess POD. A diagnosis of delirium may be made by a psychiatrist or other doctor.

### Statistical Analysis

The measurement data were expressed as the mean (standard deviation) when it was normally distributed and was described as the median (interquartile range) when it was non-normally distributed. They were processed by one-way ANOVA or Kruskal Wallis H test determined by whether they were normally distributed. The counting data were expressed as percentage or frequency, processed by chi-square test. The univariate regression model examined the correlation between each covariate and POD. Both non-adjusted and multiple-adjusted models were included in the multiple regression models. The results of unadjusted, minimally adjusted, and fully adjusted analyses were simultaneously exhibited. In the minimally adjusted model, we only adjusted for age and sex. In the fully adjusted model, we considered the covariates that might have interfered with the results based on previous literature, which included demographics (age, sex, BMI, ASA, functional status (metabolic equivalent, MET), smoking status), preoperative comorbid illness (angina, renal failure, diabetes, obstructive sleep apnea, asthma, coronary artery disease, PAH severity), arrhythmia (atrial fibrillation, atrial flutter, ventricular fibrillation, ventricular tachycardia, supraventricular tachycardia, bradycardia, heart block, premature atrial contraction, premature ventricular contraction), cardiac catheterization or echocardiogram results (right atrial systolic pressure, pulmonary arterial systolic pressure, tricuspid regurgitant velocity), preoperative medication (statin, steroids, antiplatelet, anticoagulant, hydrochlorothiazide, calcium channel blocker, beta-blocker, alpha-blocker), surgery-related factors (length of surgery, open surgical approach, intraabdominal surgery, intrathoracic surgery, vascular surgery), and intraoperative medication (sevoflurane, isoflurane, atropine). We further selected the covariates in the fully-adjusted model by the following principle: the model changed the matched odds ratio by at least 10% when adding this covariate to the model (14). According to this principle, the fully-adjusted multivariate model was adjusted for the following variables: age, BMI, smoking status, pulmonary arterial systolic pressure, length of surgery, vascular surgery, asthma, obstructive sleep apnea, renal failure, atrial fibrillation, coronary artery disease, hydrochlorothiazide, alpha-blocker, calcium channel blocker, antiplatelet, steroids, statin, and isoflurane. The R (http://www.R-project.org, The R Foundation) and EmpowerStats (http://www.empowerstats.com, X&Y Solutions, Inc., Boston, MA) statistical software was applied for data processing. *P* < 0.05 (two-sided) was considered statistically significant.

## Results

### Patient Characteristics

A total of 539 patients were included in this analysis. Baseline characteristics are listed in Table 1. 291 patients never used ACEIs or ARBs drugs, and 248 ACEIs/ARBs ever-users. The patients who never used ACEIs/ARBs were younger than the ACEIs/ARBs ever-users. The sex constituent ratios differ between the two groups. In the ACEIs/ARBs group, the proportion of males is lower. As for comorbidities, coronary artery disease and arrhythmia significantly differed between the two groups. On medication, compared with ACEIs/ARBs never-users, more ACEIs/ARBs users had taken hydrochlorothiazide, calcium channel blockers, beta-blockers, statin, and anticoagulant drugs, less ACEIs/ARBs users had taken steroids. POD occurred in 16 (3.0%) of patients. The incidence of ACEIs/ARBs users was 1.61%, while the incidence of never-users was 4.12%.

**Table 1:** Baseline characteristics.

**Variables**	**Non-ACEIs/ARBs (*n* = 291)**	**ACEIs/ARBs (*n* = 248)**	***P*-value**
**Patient characteristics**			
Age (years), mean (SD)	58.61 (14.33)	62.52 (13.35)	<0.001
**Sex**			0.039
Male	169 (58.08%)	122 (49.19%)	
Female	122 (41.92%)	126 (50.81%)	
**BMI (kg/m** ^**2**^ **), mean (SD)**	31.48 (13.27)	32.07 (10.48)	0.215
**ASA classification**			0.664
II	26 (8.93%)	17 (6.85%)	
III	198 (68.04%)	171 (68.95%)	
IV	67 (23.02%)	60 (24.19%)	
**Poor functional status (<4 MET)**	148 (50.86%)	122 (49.19%)	0.700
**Smoking status**			0.196
Never	156 (53.61%)	115 (46.37%)	
Yes	20 (6.87%)	16 (6.45%)	
Quit	115 (39.52%)	117 (47.18%)	
**Comorbidities**			
Angina	19 (6.53%)	20 (8.06%)	0.493
Renal failure	78 (26.80%)	57 (22.98%)	0.308
Diabetes	76 (26.21%)	79 (31.85%)	0.149
Obstructive sleep apnea	75 (25.77%)	67 (27.02%)	0.744
Asthma	41 (14.09%)	34 (13.77%)	0.914
Coronary artery disease	76 (26.30%)	105 (42.68%)	<0.001
Arrhythmia	108 (37.11%)	133 (53.63%)	<0.001
Bradycardia	8 (2.75%)	7 (2.82%)	0.959
Premature ventricular contraction	2 (0.69%)	5 (2.02%)	0.257
Premature atrial contraction	0 (0.00%)	1 (0.40%)	0.460
Heart block	4 (1.37%)	21 (8.47%)	<0.001
Supraventricular tachycardia	3 (1.03%)	1 (0.40%)	0.628
Ventricular tachycardia	12 (4.12%)	26 (10.48%)	0.004
Ventricular fibrillation	9 (3.09%)	14 (5.65%)	0.144
Atrial fibrillation	76 (26.12%)	83 (33.47%)	0.062
Atrial flutter	10 (3.44%)	9 (3.63%)	0.904
**PAH severity classification**			0.912
Mild	126 (44.52%)	100 (42.74%)	
Moderate	131 (46.29%)	111 (47.44%)	
Severe	26 (9.19%)	23 (9.83%)	
**Echocardiogram or cardiac catheterization data**			
RASP (mmHg), median (Q1–Q3)	5.00 (5.00–10.00)	5.00 (5.00–10.00)	0.791
PASP (mmHg), mean (SD)	44.04 (12.88)	44.76 (12.76)	0.556
TRV (m/s), mean (SD)	2.90 (2.60–3.20)	2.90 (2.70–3.26)	0.576
**Surgical characteristics**			
Length of surgery (min), median (Q1–Q3)	87.50 (34.00–152.00)	85.00 (37.00–153.00)	0.963
Intraabdominal surgery	66 (22.68%)	46 (18.55%)	0.239
Intrathoracic surgery	17 (5.84%)	10 (4.03%)	0.337
Vascular surgery	9 (3.09%)	10 (4.03%)	0.556
Open surgical approach	150 (51.55%)	134 (54.03%)	0.565
**Medications**			
Antihypertensive medication			
Hydrochlorothiazide	7 (2.41%)	36 (14.52%)	<0.001
Calcium channel blockers	43 (14.78%)	57 (22.98%)	0.015
Beta blockers	128 (43.99%)	159 (64.11%)	<0.001
Alpha blocker	10 (3.44%)	8 (3.23%)	0.892
Statin	98 (33.68%)	144 (58.06%)	<0.001
Steroids	74 (25.43%)	26 (10.48%)	<0.001
Antiplatelet	11 (3.78%)	7 (2.82%)	0.537
Anticoagulant	68 (23.37%)	79 (31.85%)	0.027
Perioperative medications			
Sevoflurane	136 (46.74%)	111 (44.76%)	0.646
Isoflurane	11 (3.78%)	7 (2.82%)	0.537
Atropine	2 (0.69%)	3 (1.22%)	0.665
**Postoperative delirium**	12 (4.12%)	4 (1.61%)	0.087

*PAH, pulmonary hypertension; RASP, right atrial systolic pressure; PASP, pulmonary arterial systolic pressure; TRV, tricuspid regurgitant velocity*.

### Univariate Analysis

The outcomes of univariate regression analysis are summarized in Supplementary Table 1. Older age (OR = 1.06, 95%CI: 1.01 to 1.11, *P* = 0.0128) and intrathoracic surgery (OR = 7.25, 95%CI: 2.17 to 24.21, *P* = 0.0013) were positively correlated with the occurrence of POD.

### The Relationship Between ACEIs/ARBs and POD

The following analysis explores the relationship between ACEIs/ARBs use and POD in multiple regression models. Results of the unadjusted and adjusted models are presented in Table 2. In the unadjusted model, the correlation of ACEIs/ARBs medication history with POD was insignificant (OR = 0.38, 95%CI: 0.12–1.20, *P* = 0.0985). This correlation approached but was insignificant in the minimally adjusted model (OR = 0.34, 95%CI: 0.10–1.10, *P* = 0.0718). However, following the adjustment of potential confounders (age, BMI, smoking status, pulmonary arterial systolic pressure, length of surgery, vascular surgery, asthma, obstructive sleep apnea, renal failure, atrial fibrillation, coronary artery disease, hydrochlorothiazide, alpha-blocker, calcium channel blocker, antiplatelet, steroids, statin, isoflurane), the fully adjusted model showed a significant negative correlation between ACEIs/ARBs use and POD (OR = 0.15, 95%CI: 0.03–0.80, *P* = 0.0266).

**Table 2:** Relationship between preoperative ACEIs/ARBs use and POD.

**Variables**	**Unadjusted model**		**Minimally-adjusted model**		**Fully-adjusted model**	
	**OR (95%CI)**	***P* value**	**OR (95%CI)**	***P* value**	**OR (95%CI)**	***P* value**
**ACEIs/ARBs**	0.38 (0.12, 1.20)	0.0985	0.34 (0.10, 1.10)	0.0718	0.15 (0.03, 0.80)	0.0266
**Age**			1.06 (1.02, 1.11)	0.0066	1.09 (1.02, 1.17)	0.0078
**Sex**			0.56 (0.18, 1.68)	0.2981		
**BMI**					1.02 (0.93, 1.11)	0.6752
**Tobacco smoking**						
Never					Ref	
Current					_ ^§^	0.9967
Former					4.35 (1.05, 18.08)	0.0432
**Hydrochlorothiazide**					_ ^§^	0.9963
**Calcium channel blocker**					3.49 (0.71, 17.22)	0.1246
**Alpha blocker**					_ ^§^	0.9970
**Renal failure**					7.42 (1.48, 37.24)	0.0149
**Obstructive sleep apnea**					0.57 (0.10, 3.35)	0.5366
**Asthma**					_ ^§^	0.9940
**Coronary artery disease**					0.06 (0.01, 0.54)	0.0114
**Atrial fibrillation**					2.87 (0.72, 11.50)	0.1355
**PASP**					1.04 (0.99, 1.10)	0.1289
**Length of surgery**					1.00 (1.00, 1.01)	0.0224
**Vascular surgery**					_ ^§^	0.9966
**Statin**					2.63 (0.63, 11.02)	0.1862
**Steroids**					0.22 (0.03, 1.54)	0.1277
**Antiplatelet**					_ ^§^	0.9969
**Isoflurane**					1.80 (0.13, 25.07)	0.6630

*Unadjusted model: We do not adjust for other covariances*.

*Minimally adjusted model: We adjust for age and sex*.

*Fully adjusted model: We included all variables in Table 1 and Supplementary Table 1 and further screened for covariates by the following principle: The model changed the matched odds ratio by at least 10% when adding this covariate. The final covariates include age, BMI, smoking status, PASP, length of surgery, vascular surgery, asthma, obstructive sleep apnea, renal failure, atrial fibrillation, coronary artery disease, hydrochlorothiazide, alpha-blocker, calcium channel blocker, antiplatelet, steroids, statin, isoflurane*.

§ *The model failed because of the small sample size*.

## Discussion

The present study examined the relationship between preoperative use of ACEIs/ARBs and POD in PAH patients. As is shown in the fully adjusted model, patients with PAH who use ACEIs/ARBs before surgery have a lower risk of POD.

A previous study showed that postoperative use of ACEIs/ARBs during intensive care was associated with lower POD odds. Furthermore, preoperative use of ACEIs/ARBs was not associated with reduced risk in that study (8). However, most formulations were relatively short-acting in that research since ACEIs/ARBs were held the morning of surgery, which may be why there was no significant difference in POD between the preoperative ACEIs/ARBs group and non-ACEIs/ARBs group (8). In addition, that study focused on patients admitted to ICU after surgery, while ICU delirium may be different from non-ICU delirium. The current study is the first to investigate the influence of preoperative ACEIs/ARBs uses on POD occurrence to the best of our knowledge.

The view of brain functional reserve can explain the protective effect of preoperative use of ACEIs/ARBs against POD. Decreased brain functional reserve is associated with the occurrence of POD (15). Thus, improving preoperative cognitive function may also have a role in preventing POD (16). Angiotensinogen in the brain, which is synthesized by the astrocytes, impairs cognitive function by increasing Aβ production, oxidative stress, inflammatory processes, and reducing acetylcholine release through AT1 receptors (7). ACEIs, e.g., perindopril, could attenuate LPS-induced cognitive impairment by suppressing oxidative stress and RAGE activation (17). Other research demonstrated that amyloidogenic processing of the amyloid precursor protein could be reduced by ACEIs treatment (18). Similar to ACEIs, ARBs have been shown to reduce cognitive deterioration by attenuating oligomerization of Aβ peptides into high molecular weight oligomeric peptides (19). Furthermore, ARBs have a protective effect on the blood-brain barrier (20). These studies indicated that ACEIs/ARBs could improve cognitive function, thereby increasing the functional reserve of the brain before surgery. These may be the possible mechanisms by which preoperative use of ACEIs/ARBs reduce POD.

This study obtained two variables associated with POD occurrence on univariate analysis. However, we are not limited to adjusting for these two variables in the fully adjusted model. In logistic regression models, OR values but not *P* values are often used to accurately represent statistical associations between risk factors and outcomes (21). In order not to omit factors that may confound the effect, we included variables that may affect POD from the original data based on previous literature and clinical experience. We screened the covariates according to the change in OR values when the variable was added and finally included covariates shown in Table 2.

Our study has some limitations. First, tools for diagnosing POD cannot be identified due to the limitations of the original database. Second, our findings are based on data from a retrospective observational study, which are inevitably prone to bias and confounders. Therefore, we used strict statistical adjustments to minimize residual confounding. Third, since the survey only includes Americans with PAH, it may not be generalized to people in other countries and patients without PAH. Finally, due to the limitations of the original data, certain pertinent variables could not be included in the analysis, such as midazolam, ketamine, dexmedetomidine, and preoperative cognitive status.

Collectively, preoperative use of ACEIs/ARBs in patients with PAH reduces the risk of POD. ACEIs/ARBs may be more recommended for patients with PAH in the future.

## Data Availability Statement

Publicly available datasets were analyzed in this study. This data can be found here: https://doi.org/10.5061/dryad.9236ng5, DATADRYAD website.

## Ethics Statement

Ethical review and approval was not required for the study on human participants in accordance with the local legislation and institutional requirements. Written informed consent for participation was not required for this study in accordance with the national legislation and the institutional requirements. Written informed consent was not obtained from the individual(s) for the publication of any potentially identifiable images or data included in this article.

## Author Contributions

GC and SZ designed the study. SZ analyzed the data. GC and FD reviewed the statistical analyses. GC wrote the manuscript. All authors read and approved the final manuscript.

## Conflict of Interest

The authors declare that the research was conducted in the absence of any commercial or financial relationships that could be construed as a potential conflict of interest.

## Publisher's Note

All claims expressed in this article are solely those of the authors and do not necessarily represent those of their affiliated organizations, or those of the publisher, the editors and the reviewers. Any product that may be evaluated in this article, or claim that may be made by its manufacturer, is not guaranteed or endorsed by the publisher.
