# Supplementary material for: Influence of Renin-Angiotensin System Inhibitors on Postoperative Delirium in Patients With Pulmonary Arterial Hypertension: A Secondary Analysis of a Retrospective Cohort Study
Source: Front Psychiatry. 2022 Apr 8;13:851104. doi: 10.3389/fpsyt.2022.851104 (PMC9024170; doi:10.3389/fpsyt.2022.851104)
Supplement: Supplementary file 1 [file Table_1.DOCX]

**Table S1: Univariate analysis**

|  | Statistics | OR (95% CI) | *P* value |
| --- | --- | --- | --- |
| Age | 60.41 ± 14.01 | 1.06 (1.01, 1.11) | 0.0128 |
| BMI | 31.75 ± 12.06 | 0.98 (0.93, 1.04) | 0.4832 |
| ASA Classification |  |  |  |
| II | 43 (7.98%) | ref |  |
| III | 369 (68.46%) | _ § | 0.9880 |
| IV | 127 (23.56%) | _ § | 0.9875 |
| Sex |  |  |  |
| Male | 291 (53.99%) | ref |  |
| Female | 248 (46.01%) | 0.52 (0.18, 1.53) | 0.2366 |
| Poor functional status (<4 MET) |  |  |  |
| No | 269 (49.91%) | ref |  |
| Yes | 270 (50.09%) | 2.24 (0.77, 6.54) | 0.1394 |
| Tobacco smoking |  |  |  |
| Never | 271 (50.28%) | 1.0 |  |
| Current | 36 (6.68%) | 1.52 (0.17, 13.39) | 0.7060 |
| Former | 232 (43.04%) | 2.40 (0.81, 7.11) | 0.1154 |
| ACEIs/ARBs |  |  |  |
| No | 291 (53.99%) | ref |  |
| Yes | 248 (46.01%) | 0.38 (0.12, 1.20) | 0.0985 |
| Hydrochlorothiazide |  |  |  |
| No | 496 (92.02%) | ref |  |
| Yes | 43 (7.98%) | _ § | 0.9878 |
| Calcium channel blocker |  |  |  |
| No | 439 (81.45%) | ref |  |
| Yes | 100 (18.55%) | 1.48 (0.47, 4.70) | 0.5032 |
| Beta blocker |  |  |  |
| No | 252 (46.75%) | ref |  |
| Yes | 287 (53.25%) | 0.87 (0.32, 2.37) | 0.7917 |
| Alpha blocker |  |  |  |
| No | 521 (96.66%) | ref |  |
| Yes | 18 (3.34%) | _ § | 0.9922 |
| Angina |  |  |  |
| No | 500 (92.76%) | ref |  |
| Yes | 39 (7.24%) | 0.85 (0.11, 6.62) | 0.8774 |
| Renal failure |  |  |  |
| No | 404 (74.95%) | ref |  |
| Yes | 135 (25.05%) | 1.37 (0.47, 4.03) | 0.5625 |
| Diabetes |  |  |  |
| No | 383 (71.19%) | ref |  |
| Yes | 155 (28.81%) | 1.13 (0.39, 3.30) | 0.8269 |
| Obstructive sleep apnea |  |  |  |
| No | 397 (73.65%) | ref |  |
| Yes | 142 (26.35%) | 0.64 (0.18, 2.27) | 0.4873 |
| Asthma |  |  |  |
| No | 463 (86.06%) | ref |  |
| Yes | 75 (13.94%) | _ § | 0.9896 |
| Coronary artery disease |  |  |  |
| No | 354 (66.17%) | ref |  |
| Yes | 181 (33.83%) | 0.48 (0.13, 1.72) | 0.2607 |
| Arrhythmia |  |  |  |
| No | 298 (55.29%) | ref |  |
| Yes | 241 (44.71%) | 1.61 (0.59, 4.40) | 0.3502 |
| Atrial fibrillation |  |  |  |
| No | 380 (70.50%) | ref |  |
| Yes | 159 (29.50%) | 2.46 (0.91, 6.68) | 0.0766 |
| Atrial flutter |  |  |  |
| No | 520 (96.47%) | ref |  |
| Yes | 19 (3.53%) | _ § | 0.9919 |
| Ventricular fibrillation |  |  |  |
| No | 516 (95.73%) | ref |  |
| Yes | 23 (4.27%) | _ § | 0.9911 |
| Ventricular tachycardia |  |  |  |
| No | 501 (92.95%) | ref |  |
| Yes | 38 (7.05%) | 0.88 (0.11, 6.81) | 0.8991 |
| Supraventricular tachycardia |  |  |  |
| No | 535 (99.26%) | ref |  |
| Yes | 4 (0.74%) | _ § | 0.9913 |
| Bradycardia |  |  |  |
| No | 524 (97.22%) | ref |  |
| Yes | 15 (2.78%) | _ § | 0.9890 |
| Heart block |  |  |  |
| No | 514 (95.36%) | ref |  |
| Yes | 25 (4.64%) | _ § | 0.9907 |
| Premature atrial contraction |  |  |  |
| No | 538 (99.81%) | ref |  |
| Yes | 1 (0.19%) | _ § | 0.9934 |
| Premature ventricular contraction |  |  |  |
| No | 532 (98.70%) | ref |  |
| Yes | 7 (1.30%) | _ § | 0.9925 |
| RASP | 8.18 ± 4.75 | 0.94 (0.82, 1.08) | 0.3845 |
| PASP | 44.37 ± 12.82 | 1.01 (0.98, 1.05) | 0.4077 |
| TRV | 3.01 ± 1.42 | 1.05 (0.83, 1.32) | 0.7051 |
| PAH Severity Class |  |  |  |
| Mild | 226 (43.71%) | ref |  |
| Moderate | 242 (46.81%) | 1.71 (0.56, 5.17) | 0.3443 |
| Severe | 49 (9.48%) | 1.88 (0.35, 9.99) | 0.4584 |
| Length of surgery | 113.82 ± 109.99 | 1.00 (1.00, 1.01) | 0.0581 |
| Intraabdominal surgery |  |  |  |
| No | 427 (79.22%) | ref |  |
| Yes | 112 (20.78%) | 0.54 (0.12, 2.39) | 0.4145 |
| Intrathoracic surgery |  |  |  |
| No | 512 (94.99%) | ref |  |
| Yes | 27 (5.01%) | 7.25 (2.17, 24.21) | 0.0013 |
| Vascular surgery |  |  |  |
| No | 520 (96.47%) | ref |  |
| Yes | 19 (3.53%) | _ § | 0.9919 |
| Open surgical approach |  |  |  |
| No | 255 (47.31%) | ref |  |
| Yes | 284 (52.69%) | 1.51 (0.54, 4.23) | 0.4280 |
| Statin |  |  |  |
| No | 297 (55.10%) | ref |  |
| Yes | 242 (44.90%) | 1.60 (0.59, 4.36) | 0.3581 |
| Steroids |  |  |  |
| No | 439 (81.45%) | ref |  |
| Yes | 100 (18.55%) | 0.62 (0.14, 2.77) | 0.5309 |
| Antiplatelet |  |  |  |
| No | 521 (96.66%) | ref |  |
| Yes | 18 (3.34%) | _ § | 0.9922 |
| Anticoagulant |  |  |  |
| No | 392 (72.73%) | ref |  |
| Yes | 147 (27.27%) | 1.63 (0.58, 4.56) | 0.3554 |
| Sevoflurane |  |  |  |
| No | 292 (54.17%) | ref |  |
| Yes | 247 (45.83%) | 1.19 (0.44, 3.21) | 0.7340 |
| Isoflurane |  |  |  |
| No | 521 (96.66%) | ref |  |
| Yes | 18 (3.34%) | 1.98 (0.25, 15.90) | 0.5187 |
| Atropine |  |  |  |
| No | 530 (99.07%) | ref |  |
| Yes | 5 (0.93%) | _ § | 0.9903 |

§ The model failed because of the small sample size.
